# Robots with Display Screens: A Robot with a More Humanlike Face Display Is Perceived To Have More Mind and a Better Personality

**DOI:** 10.1371/journal.pone.0072589

**Published:** 2013-08-28

**Authors:** Elizabeth Broadbent, Vinayak Kumar, Xingyan Li, John Sollers, Rebecca Q. Stafford, Bruce A. MacDonald, Daniel M. Wegner

**Affiliations:** 1 Department of Psychological Medicine, University of Auckland, Auckland, New Zealand; 2 Department of Electrical and Computer Engineering, University of Auckland, Auckland, New Zealand; 3 Psychology Department, Harvard University, Cambridge, Massachusetts, United States of America; ICREA-University of Barcelona, Spain

## Abstract

It is important for robot designers to know how to make robots that interact effectively with humans. One key dimension is robot appearance and in particular how humanlike the robot should be. Uncanny Valley theory suggests that robots look uncanny when their appearance approaches, but is not absolutely, human. An underlying mechanism may be that appearance affects users’ perceptions of the robot’s personality and mind. This study aimed to investigate how robot facial appearance affected perceptions of the robot’s mind, personality and eeriness. A repeated measures experiment was conducted. 30 participants (14 females and 16 males, mean age 22.5 years) interacted with a Peoplebot healthcare robot under three conditions in a randomized order: the robot had either a humanlike face, silver face, or no-face on its display screen. Each time, the robot assisted the participant to take his/her blood pressure. Participants rated the robot’s mind, personality, and eeriness in each condition. The robot with the humanlike face display was most preferred, rated as having most mind, being most humanlike, alive, sociable and amiable. The robot with the silver face display was least preferred, rated most eerie, moderate in mind, humanlikeness and amiability. The robot with the no-face display was rated least sociable and amiable. There was no difference in blood pressure readings between the robots with different face displays. Higher ratings of eeriness were related to impressions of the robot with the humanlike face display being less amiable, less sociable and less trustworthy. These results suggest that the more humanlike a healthcare robot’s face display is, the more people attribute mind and positive personality characteristics to it. Eeriness was related to negative impressions of the robot’s personality. Designers should be aware that the face on a robot’s display screen can affect both the perceived mind and personality of the robot.

## Introduction

### Motivation

As technology advances, socially assistive robots are being developed for healthcare contexts [1], [2]. It is important that such robots have an appearance that users feel comfortable with [3]. Practically, robots cannot be completely humanlike at the current stage of robotics development. While robots such as HRP4 have been developed [4], they are too expensive and not capable of deployment in real world scenarios. Furthermore, interactions in speech are not easy, and vision for interaction is not very technically feasible. More practical robots have wheels and interact with touchscreens and speech output. Some have faces presented on the touchscreen and some do not have any faces; others have simplified plastic faces that have some lighting effects, and some have moving parts. It is important to understand how people perceive different robot designs and help designers create appropriate robots. For touchscreen robots, it is important to know whether to put faces on the screen to enhance the interactions and what kinds of faces are better than others. Examples of healthcare robots with touchscreen displays include the Healthbot and Care-o-bot [5], [6]. This study aimed to understand robots with screens, and to give developers advice about the design of faces for screen display. Our goal was to evaluate the kind of face that a display screen robot might best show, and whether having a face on the screen is important. We hypothesize that the results will be translatable to real 3D versions of the faces displayed but we do not evaluate this claim in this paper. We seek to establish some knowledge based on screen-displayed faces in order to discover important factors, which is necessary before more expensive real faces are constructed since the practicalities of constructing real 3D faces are considerable. Since we typically interact with humans but we cannot yet make completely humanlike robots, a very relevant factor in the design of robot faces is the degree of humanlike appearance, and this is the focus of the current study. The introduction reviews previous work in the area of humanlike appearance on robots, including theories behind the effects observed. It then goes on to introduce the current study.

### Humanlike Appearance of Robots

One theoretical model of the relationship between robot appearance and how comfortable humans feel with it is The Uncanny Valley theory [7], [8], which proposes that as robots become more humanlike, we feel more comfortable with them. But when a robot becomes highly humanlike we still know there is something that is not real about it, and then we experience a sense of strangeness or eeriness. The uncanny valley has been likened to how we might feel about seeing a dead person, and has implications for the design of acceptable robots. While the Uncanny Valley is popular, there has been little empirical research to test the theory. However, more work has been done over the past ten years. A recent review concluded that evidence for the Uncanny Valley is equivocal [9]. In a case study, only a few people mentioned that a Geminoid robot (very humanlike but not human) gave them an uneasy feeling [10].

Humanlikeness can refer not only to visual appearance but also to behavioral features, such as the voice and movement of a robot. Research has shown that robots with humanlike voices are rated more highly than those with an identical appearance but with more a robotic voice [11], [12]. Other work has shown that a mismatch between human or machinelike *appearance* with human or machinelike *voice* creates eeriness [13]. Research on movement has shown that avatars that move in a more humanlike manner are perceived as more familiar and less eerie [14]. However, there is some preliminary evidence for uncanny effects when mechanical movement is paired with an android robot [15].

Research on the Uncanny Valley and facial appearance has evaluated images of robots on scales that rate appearance (e.g. humanlike, familiar, eerie, appeal, creepy, strange), and emotional reactions (e.g. fear, disgust) [16], [17]. Recent work asked people to rate a series of photographs that morphed a robot’s photo to a human’s photo, and found that there was a dip in familiarity that corresponded to a peak in eeriness in the middle of the series [18]. Feelings of eeriness have been shown to disappear when the morphing is more carefully crafted, suggesting poor mixing of the faces rather than the humanlikeness of the image may be the cause of perceived eeriness [16]. Indeed, research has suggested that the mixture of robot and human features may be the cause of perceived eeriness [17]. In line with this, robots that have a clearly non-human appearance are liked more than robots that try to appear humanlike [19]. Other work has shown that a bronze face with simplified eyes is rated more eerie than both a photorealistic face and a line drawing face [8].

### Theories behind the Effects of Humanlike Appearance of Robots

Some work suggests that the Uncanny Valley may have an evolutionary purpose to cause animals to avoid unhealthy individuals. Monkeys spent longer looking at real or unrealistic synthetic faces than looking at realistic synthetic faces [20]. The authors suggest that the monkeys identified the realistic synthetic faces as belonging to their own species more than the unrealistic synthetic faces, but these faces failed to live up to expectations for appearance and behavior. Other studies suggest that there is a developmental basis to Uncanny Valley effects, which requires early experience with real faces, as infants do not exhibit such effects until the age of 12 months [21].

A number of theories are relevant to the Uncanny Valley. One prominent theory is expectation violation. A humanlike appearance can induce expectations of humanlike behavior. When a robot looks human but does not behave in a humanlike manner, this violates expectations and can lead to surprise or fright [13], [18]. It has been shown that people come to interactions with pre-existing mental representations of what robots can do and look like [19]. These expectations can influence reactions to the robot. For example, using the same robot as used here (with a simple humanlike face on the screen), a previous study showed that people who had pre-existing ideas that a healthcare robot would be humanlike had higher blood pressure during the interaction than those who imagined the robot would be more machine-like [22]. In related theory, Bayesian modeling posits that when there are conflicting cues as to which category an object belongs to, this can create perceptual tension, which in turn promotes behavior to decrease this tension – such as withdrawal, attack, ignoring one of the cues, or attempts to reduce the misalignment between categories [23]. It has been suggested that faces can be categorized as human or non-human and where there is ambiguity this creates discomfort [24]. Effectance motivation describes the desire to interact effectively with the environment, and to understand, predict and have control over it. It has been proposed that anthropomorphism of robots increases our feelings of understanding and control over them and reduces uncertainty [25]. A humanlike appearance may therefore increase acceptance of the robot.

### Mind perception and Personality

A recently proposed theory about why humanlike robots can appear uncanny is that humanlike features prompt users to perceive that the robot has a mind [26]. This is related to anthropomorphism, in that a humanlike appearance prompts expectations of humanlike attributes. In the theory of mind, humans ascribe minds to other people in order to establish common ground and to enable communication. It has been suggested that developers should build a theory of mind into agents to enable better communication [27]. Research in social psychology has investigated the dimensions along which we judge whether something has a mind and developed the Mind Perception Questionnaire to assess these dimensions [28]. This research has shown two dimensions of mind perception – agency (the capacity to do things) and experience (the capacity to experience things). Both dimensions are associated with the liking of a character. The capacity for experience has been linked to being afforded moral rights and the capacity for agency has been linked to having moral responsibility [28]. It has been suggested that the concept of agency (having the ability to interact, be autonomous, and adaptable) allows computers and animals to be considered moral agents without needing to have free will, emotions or mental states [29]. In an initial survey, people rated a robot very low in the capacity to experience but with a moderate amount of agency [28]. The robot was rated lower in both experience and agency than a human adult, but higher in agency than a baby, a monkey and a dog. Of all the other rated ‘beings’ in the study, a dead woman was rated closest to a robot in this two dimensional space. It has been proposed that the uncanny valley may be caused by the perception that a being has an incomplete mind, e.g. the robot has the capacity to do but not feel [30].

Studies using this Mind Perception Questionnaire have looked at how varying the humanness of a robot’s voice can affect perceptions of robot mind [31]. Results suggested that female participants attributed more mind to a female voiced robot and males attributed more mind to a male voiced robot. That study summed all items rather than use the two subscales published in the original paper. Other work has examined appearance; participants who viewed a video of the robot Kaspar from the front (more humanlike) compared to the back (more mechanical), attributed a greater capacity to experience to the robot, and this capacity was linked to perceptions of the robot being uncanny [26]. However, the front and back viewpoint may have contributed to the difference in perceptions rather than humanlikeness per se, and the participants watched a video rather than interacting with a physically present robot. Further research is needed to investigate how the humanlikeness of a robot is linked to perceptions of its mind and how this relates to eeriness in uncanny valley theory in a human-robot interaction with different kinds of faces presented from the front.

Another reason why humanlike robots are seen as uncanny may be that humanlike facial features cause the user to perceive that the robot has certain personality traits that cause feelings of unease. In humans, facial appearance has been shown to affect perceptions of a person’s personality [32]. Similarly, the degree of humanlikeness of a robot’s face may also affect perceptions of the robot’s personality. Previous work supports this theory. A robot’s facial features, such as a nose, mouth and eyebrows, can contribute to ratings of how humanlike a robot is [31], [33]. There is evidence that the shape of a robot’s head can influence ratings of the robot’s knowledge and sociability [34]. Children rate robots to have different amounts of happiness and sadness, as well as different degrees of behavioural intention, based on their appearance [35]. Other research has shown that more mechanical robots are rated to be lower on emotional stability, extroversion, agreeableness, conscientiousness and intellect, than a humanoid robot [36]. A robot with a higher-pitched voice is perceived to have a different personality to a robot with a lower pitched voice [37]. Avatars have also been rated differently on personality compared to agents [38].

### Summary and Current Study

This review of previous work has shown that there are many aspects of a robot’s appearance that can affect how the robot is perceived, and a number of possible theories behind these effects. The current study aimed to investigate the effects of humanlike facial appearance on perceptions of the robot’s eeriness, mind, and personality, as well as on the participants’ blood pressure. It also explored whether personality and mind were correlated with eeriness. We specifically aimed to investigate these effects on a robot with a display screen because robots with interactive touch screens are becoming more popular for practical applications in real world scenarios. We hypothesised that a robot with no-face at all on its display screen would be perceived as being the least alive and having the least mind; a robot with a humanlike face on the display screen would be perceived as having the most mind, and being most alive. However, a robot with a silver face on its display screen would be seen as intermediate in mind and aliveness, the face would be rated as more eerie, and people would have greater blood pressure (reflecting their unease). We hypothesised that the robot with the humanlike face display would be seen most positively in terms of personality.

## Materials and Methods

### Ethics Statement

The study was granted ethical approval by the University of Auckland Human Participants Ethics Committee. All participants provided written informed consent.

### Participants

Thirty participants (14 female and 16 male) completed the study. Participants were recruited from email advertisements to university students and staff. The participants had a mean age of 22.50 years (range 18–38, SD 4.58). Inclusion criteria were age over 16 years and able to understand English.

### The Robot

**Figure 1:**
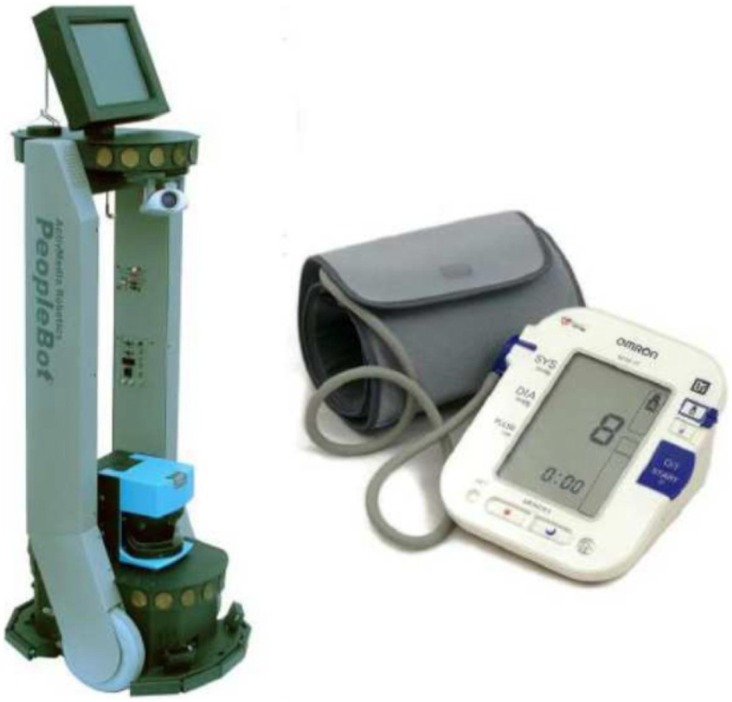
Peoplebot robot and blood pressure monitor that was attached to it for the study.

In this experimental scenario, a Peoplebot robot with an on board Intel Pentium1300 MHz processor was used to act the part of a helper for a human nurse. Peoplebot is produced by Adept Mobile Robots (USA) and is designed for service and human-robot interaction projects. The robot was equipped with a speaker to talk, a display to show its face and a cuff blood pressure monitor connected to the robot via USB to measure the patient's blood pressure. Dialogues were designed for the robot to instruct the participant to use the blood pressure monitor, display the result on its screen and verbally report the results verbally (see Figure 1). The robot can move forward and rotate but has no articulated parts. This scenario has been used in previous studies [11], [39].

**Figure 2:**
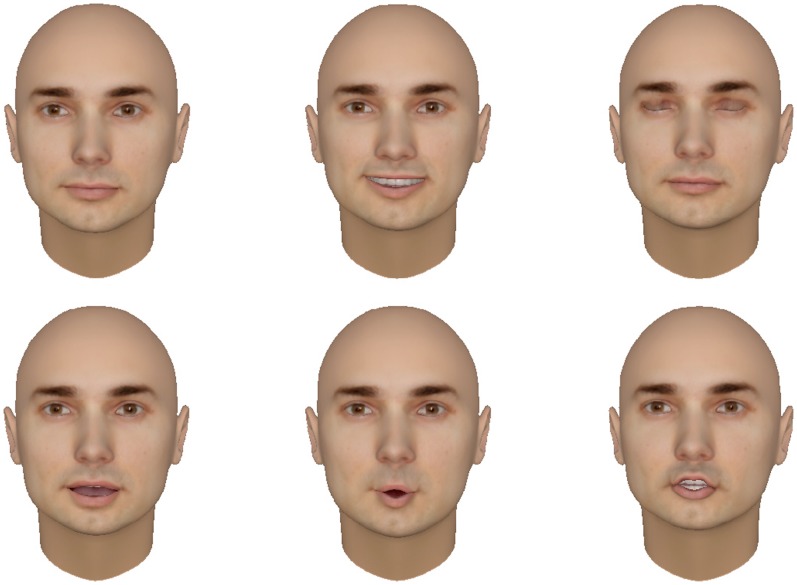
The human-like face created by Facegen and how the 3D face looks with difference expressions and modifiers. Top row: normal face, smile, blink. Bottom row: speak “Ah”, speak “Oh”, speak “J”.

The robot’s display shows a 3D virtual face, which is capable of expressing several emotions as well as rendering the correct lip movements for speech. The virtual face has over 6000 polygons and looks humanlike. Software FaceGen Modeller, from Singular Inversions, can generate realistic 3D faces randomly or from a real person’s photograph. The face model can be any race, gender or adult age with different expressions, phonemes and modifiers. It allows the user to control the texture color, symmetric shape and add extra parts such as eyeglasses or hats. To animate the virtual face model so that it speaks in a natural way, we use Xface, an open source 3D talking head based on the MPEG-4 standard. The Xface toolkit is optimized enough to achieve at least 25 frames per second with a polygon count up to 12000, using modest hardware. To create the faces, a photograph was taken of a male student volunteer of European ethnicity. Hair was removed in the software. Figure 2 shows the 3D virtual face created by Facegen and how the 3D face looks with difference expressions and modifiers.

**Figure 3:**
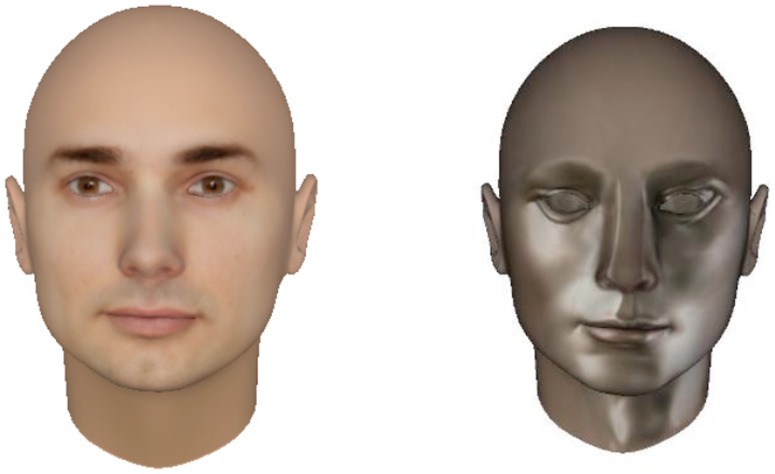
The silver face (right), modifed from the humanlike face by changing the skin texture and colour, and the eyes.

The second face was created to look more robotic. The shape (mesh) of the first face was used and only its appearance was modified. As Figure 3 shows, the humanlike skin was replaced with the silver metal-like surface and the human eyes were replaced with blank holes to simplify them. Both faces had the same animation quality because they both used X-face animation software.

### Procedure

Each participant was invited to interact with the robot three times. They were told that the aim of the study was to see which robot people preferred. Each time the robot performed the same task: to enter the room, move to the participant, and assist the participant in taking their blood pressure. In each interaction the robot had a different face displayed on its screen: no-face display (NFD), a humanlike face display (HFD) or a silver face display (SFD). The NFD robot had the words “healthcare robot” on the screen instead of a face. The order of the robots with the different screen displays was randomly assigned and counter-balanced for each participant.

At the start of the interaction, the robot introduces itself to the participant and asks them how they are today, and tells them that it would like to measure their blood pressure. It asks the participant to roll up the sleeve of either arm, and to undo the velcro fastening on the cuff and to slide their arm into it making sure that the cord is on the inside part of the arm and coming out of the bottom of the cuff, and to refasten the cuff. At that point, the robot displays a video showing them how to do this. The robot asks them to press the start button on the meter when they are ready and tells them that the cuff will automatically inflate and deflate of its own accord, and when it is finished the robot will tell them the results. The robot then tells them that they have done very well and says their blood pressure and heart rate results. The results are displayed on the robot’s screen underneath the face. The robot then thanks the participant and moves out of the room. After each interaction, the participant completed a questionnaire.

### Measures

The questionnaire contained a 6-item version of the Mind Perception Questionnaire [28]. The scale contains two subscales – agency (the ability to think, recognise emotions, communicate) and experience (the ability to feel pleasure, pain, be conscious). Participants were asked to “Please indicate the degree to which you believe this robot has each of these capacities:” on scales from 1 to 7. For example, “How much is this robot capable of thinking?” Scores were summed for each subscale where a higher score indicated greater capacity (possible range 3 to 21 for each subscale). The scales were internally reliable for all faces (agency range.76 to.85; experience range.70 to.85).

The questionnaire also contained three visual analog scales asking the participants: “How humanlike did you think this robot was?” (very machine-like ‘0’ to very humanlike ‘100’); “Did the robot seem alive?” (not at all alive ‘0’ to very much alive ‘100’), and “How eerie did the robot’s face look?” (not at all eerie ‘0’ to very eerie ‘100’, for the human and silver faces only).

For each face display condition, participants rated their impression of the robot’s personality using Asch’s checklist of characteristics [40]. This list comprises 18 pairs of traits, mostly opposites, and participants are asked to “Please select one word from each pair that is most in accordance with the view you have formed of this robot:”. The pair warm-cold was added due to evidence of its primary importance in Asch’s paper. After recoding negatively valenced items, factor analysis with varimax rotation was used to discern personality factors using the humanlike face display condition, and a scree plot indicated three factors explained the majority of variance. Factor one “sociable” included six items: unsociable-sociable, unpopular-popular, hard headed-imaginative, cold-warm, humourless–humourous, and irritable-good natured, (cronbach’s alpha.82). Factor two “amiable” included four items: unattractive-good looking, unhappy-happy, ruthless-humane, and ungenerous-generous, (cronbach’s alpha.76.) The third factor “trustworthy” included three items: unstable-persistent, shrewd-wise and dishonest-honest (cronbach’s alpha.63). Subscales were created to represent these factors by summing these items for each face. Participants were also asked to “Please give a brief characterization of this robot in just a few sentences:”. After interacting with all three robot face display conditions, the participants completed a final questionnaire in which they were asked, “Please rank below, which robot you liked the most for a healthcare robot, from most favorite to least favorite”.

Data were analysed using SPSS version 19. One sample Pearson’s chi-square was used to analyse robot face display condition preference. Repeated measures ANOVA were used to compare differences in ratings between the three conditions, with post-hoc tests using Sidak’s adjustment for multiple comparisons. Pearson’s correlations were run to investigate how perceptions of mind were related to how humanlike, alive and eerie each condition was rated. Associations between eeriness and personality factors were conducted using Pearson’s r. Significance was set at p<.05.

## Results

### Robot Face Display Preference

When asked which was their favourite healthcare robot, 18 participants (60%) chose the robot with the HFD, nine chose the robot with the NFD (30%) and three (10%) chose the robot with the SFD, *χ^2^* (2, *N* = 30) = 11.40, *p* = .003.

### Differences between Robot Face Display Conditions in Ratings of Humanlike, Alive, and Eerie

The robots with the three different face displays were rated significantly differently on humanlikeness *F* (2, 29) = 25.00, *p*<.001. The humanlikeness means were: NFD 21.70 (*SD* 16.10); SFD 39.20 (*SD* 18.80); HFD 49.37 (*SD* 23.76). Post–hoc tests indicated the NFD condition was rated significantly less humanlike than both the SFD condition (*p*<.001), and the HFD condition (p<.001). In addition, the HFD condition was rated significantly more humanlike than the SFD condition (*p* = .020) There was also a significant difference in ratings of being alive, with perceived aliveness increasing with humanlikeness of the face display conditions; *F* (2, 29) = 10.63, *p*<.001. The alive rating means were: NFD 20.47 (*SD* 20.13); SFD 31.30 (*SD* 22.77); and HFD 40.63 (*SD* 26.16). Post-hoc tests indicated that there was a significant difference between NFD and HFD conditions (*p*<.001), but the difference between the NFD and SFD conditions was not significant (*p = *.057), and the difference between the SFD and HFD conditions was also not significant (*p* = .055). The SFD was rated more eerie (mean 54.47, SD 24.17) than the HFD condition (mean 39.70, *SD* 25.99), *t* (29) = 2.46, *p = *.020.

### Differences between Robot Face Display Conditions in Perceived Agency and Experience

**Figure 4:**
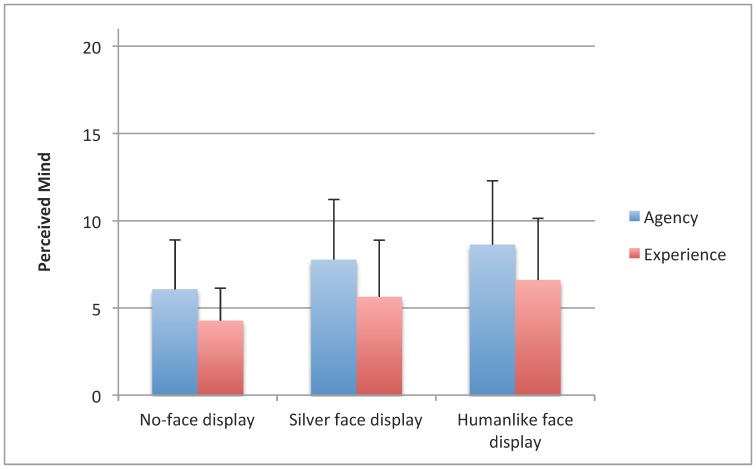
Differences in perceived agency and experience of the robot between the different face conditions (mean, *SD*).

There was a significant difference in ratings of how much agency each robot had depending on face display condition, *F* (2, 58) = 9.34 (*p*<.001), see Figure 4. Post-hoc tests showed significant differences between the NFD and the SFD conditions (*p* = .049) and between the NFD and the HFD conditions (*p*<.001), but the difference between the SFD and HFD conditions was not significant (*p* = .259). Similarly, there was a significant difference in ratings of how much each robot could experience things depending on its face screen display *F* (2, 58) = 11.20, *p*<. 001, see Figure 4. Post hoc tests showed significant differences in experience between NFD and the SFD conditions (*p* = .022), and between NFD and the HFD conditions (*p* = .001) but the difference between the SFD and HFD conditions was not statistically significant (*p* = .072).

### Differences between Robot Face Display Conditions in Personality Impressions

There were significant overall differences between robots with different face displays for sociability and amiability but there was no significant difference in the trustworthy factor between the robot face display conditions (see Table 1). Post-hoc tests with Sidak’s correction indicated a significant difference in sociability between the NFD and the HFD conditions (*p = *.011), while the difference between NFD and the SFD conditions was not significant (*p* = .069), and neither was the difference between the SFD and the HFD conditions (*p* = .315). There was a significant difference between the HFD and NFD conditions in amiability (*p* = .001) and between the HFD and the SFD conditions (*p* = .025), but the difference between the SFD and NFD conditions was not significant (*p* = .196).

**Table 1:** Personality ratings of the robot: differences between face display conditions. Overall *F* and *p* value are shown.

PersonalityFactor	Possiblerange	No-face displayMean (SD)	Silver face displayMean (SD)	Humanlike facedisplay Mean (SD)	*F(2,58)*	*p* value
1. Sociable	0–6	1.50 (1.25)	2.17 (1.91)	2.57 (1.98)	4.034	.023
2. Amiable	0–4	1.50 (1.41)	1.93 (1.48)	2.63 (1.45)	6.74	.002
3. Trustworthy	0–3	2.70 (0.47)	2.63 (0.55)	2.57 (0.77)	0.66	.521

Some examples of the brief personality impressions people gave for each robot are listed here. HFD: “Felt more close to the robot. It felt affectionate and humanlike. The face looked like a real doctor’s face serious/reliable”, “Robot seemed friendly, helpful, patient – didn’t rush”, “practical, calm, predictable, reliable, task-oriented, efficient”. SFD: “I thought it was efficient however it did lack some emotion…”, “Practical…sterile…”, “enough humanlike that the silver appearance becomes creepy”. NFD: “very robotic, impersonal, mechanical and soulless”, “…the robot does not give warmth and does not comfort you”, “…In some ways more honest because there isn’t a face and I know there is not a person inside…”.

### Differences between Robot Face Display Conditions in Blood Pressure

The repeated measures ANOVA showed no overall significant effect of robot face display condition on diastolic (*F* (3, 87) = 0.40, *p = *.750), or systolic blood pressure (*F* (3, 87) = 0.90, *p = *.446).

### Associations between Perceptions of having a Mind, being Alive, Humanlike, and Eerie

For each robot face display condition, there was a strong correlation between ratings of humanlikeness and being alive: NFD *r* = .73 (*p*<. 001), SFD *r* = .76 (*p*<. 001) and HFD *r* = .77 (*p*<. 001). Ratings of humanlikeness were significantly related to agency (NFD *r* = .37 (*p* = .046), SFD *r* = .36 (*p* = .048), HFD *r* = .68, *p*<.001), and to perceived capacity to experience for the HFD only (*r* = .59, *p*<.001). Ratings of humanlikeness were not significantly related to perceived capacity to experience for the NFD (*r* = .34, *p* = .066) or SFD conditions (*r* = .13, *p* = 483).

Ratings of being alive were related to agency ratings for both the SFD (*r* = .62, *p*<.001), and HFD conditions (*r* = .67, *p*<.001), and with the capacity to experience for both SFD (*r* = .41, *p* = .023), and HFD conditions (*r* = .50, *p* = .005). There were no significant relationships between ratings of being alive and agency or experience for the NFD condition (*r* = .108, *p* = .571), and *r* = .039 (*p* = .838) respectively.

The eeriness of the SFD and HFD conditions was not significantly related to ratings of being alive (SFD *r* = −.002, *p* = .990; HFD *r* = .11, *p* = .557), nor to being humanlike (SFD *r* = −.14, *p* = .458; HFD *r* = −.30, *p* = .110), nor to perceptions of agency (SFD *r* = .15, *p* = .437, and HFD *r* = −.21 *p* = .261) or experience (SFD *r* = −.004, *p* = .984, and HFD *r* = −.208, *p* = .271).

### Associations between Eeriness and Personality

For the robot with the HFD, there were significant correlations with eeriness for all three factors: sociable (*r* = −.517, *p* = .003), amiable *(r* = −.535, *p* = .002), and trustworthy (*r* = −.514, *p* = .004). For the robot with the SFD, these correlations did not reach significance (*r* = −.148, *p* = .438), amiable (*r* = −.242, *p* = .197) and trustworthy (*r* = .224, *p* = .235).

## Discussion

This study presented three different faces on a robot with a display screen – no-face display, a silver face display, and a humanlike face display. As expected, the robot with the humanlike face display was rated the most humanlike, followed by the robot with the silver face display, and then the robot with the no-face display. The more humanlike the face display, the more people attributed mind (the ability to experience things and have agency) to the robot, and the more they saw the robot as being alive. The face displays influenced impressions of the robot’s personality, with the humanlike face display seen as the most sociable and amiable, but all three were seen as trustworthy. The robot with the humanlike face display was the most preferred, followed by the robot with the no-face display, and the robot with the silver face display was the least preferred. The robot with the silver face display was rated more eerie than the robot with the more humanlike face display. There were no differences in participants’ blood pressure between robot face display conditions.

The results are in line with Eyssel’s theory that people seek to attribute humanlike qualities to robots [25], and suggest that a humanlike appearance may augment this process. The findings tie in with the expectation violation and Bayesian models, because one interpretation is that the silver face provides conflicting cues as to whether the face is human or artificial. Mind perception processes may be part of this model, such that robots may not be expected to be able to feel emotions – but humans are, and the silver face could be seen to provide conflicting cues as to which category the entity belongs.

These results are congruent with initial studies on mind perception that have shown people attribute more mind to adult humans than all other characters [28]. They also align with results that people attribute more mind to robots with voices of the their own gender [31], and this indicates that people attribute more mind to others that are more similar to themselves. This is the first study to show that changing the face can influence how much mind people attribute to a robot with a display screen during an interaction. The study used Mind Perception Questionnaire, which breaks mind into the capacity for experience and the capacity for agency. The advantage of this questionnaire is that is has been psychometrically validated, has distinct subscales for agency and experience, and has been used in different contexts and so allows comparisons to a range of other entities.

Designers need to think carefully about what qualities they wish their robot to be perceived as having and design the face accordingly. A humanlike face display should be used if the designers wish the robot to be perceived as having greater abilities to experience things, have agency and be seen as more sociable and amiable. On other hand if designers do not want people to have high expectations of the robot having these abilities, then a humanlike face display may not be useful. For example, retirement village residents have expressed a preference for a robot that is not humanlike [41], and they may not want a robot that they perceive can think and feel. The face on a robot presents information from which people form impressions and expectations of abilities. Previous research has found that users’ overly-high expectations of a robot’s abilities before an interaction are adjusted down after the interaction, while overly low expectations are adjusted up, suggesting that it is better to create low expectations to avoid disappointment [42].

The robot with the humanlike face display was rated highest in the capacity to experience followed by the robot with the silver face display, and then the no-face display, which is similar to findings that the front of a robot’s head is rated higher in experience than the back [26]. This study found that the robots with the humanlike and silver face displays were rated as having greater agency than the no-face display, whereas the front and back of the head were rated as similar in agency in Gray and Wegner’s experiment. These new findings suggest that the presence of even a silver face display can promote perceptions of agency compared to no-face display.

Interestingly, ratings of eeriness were not related to ratings of humanlikeness or being alive. This finding supports earlier work that it is not the degree of humanlikeness per se that creates eeriness [16], [43]. Instead, ratings of eeriness were significantly associated with the impression of personality; in particular, higher eeriness was related to perceptions of being less sociable, amiable, and trustworthy for the robot with the humanlike face display. The open–ended descriptions of personality suggested that people inferred warmth, affection, and friendliness from appearance. All of the faces were rated highly on trustworthiness, which is a characteristic that is desirable for a healthcare robot.

Neither ratings of experience nor agency were significantly correlated with perceptions of eeriness, whereas in earlier work, the capacity of experience partially explained feelings of uncanniness [26]. These differences in findings may be due to methodological factors – this study used a within-group design rather than between groups design, all of the participants viewed the robot from the front, the robot was a Peoplebot rather than Kaspar, participants interacted with a robot with a display screen rather than watching a video of a robot, and there were differences in questionnaire items. The previous study assessed perceptions of the robots’ ability to experience pain and fear, whereas this study assessed perceptions of the robots’ ability to experience pain, pleasure and consciousness. The findings of this paper suggest that the perceived capacity to experience per se is not as important to the Uncanny Valley as the perceived lack of sociability and amiability. However, more research is needed to explore this further.

Limitations of the study include the presentation of a limited number of faces, and only the one robot body type. Using blank holes on the silver face may not have been the best method to simplify the eyes, however, we were limited by the software capabilities. Future research could further investigate how other forms of face and body affect perceptions of personality and mind and link to the Uncanny Valley. It could be argued that it is not clear whether the participants were rating the faces on the display screens or the robots as a whole. We argue that the participants saw each screen display as part of each robot, so they were in effect rating the robots as a whole. In support of this point: the questionnaires specifically asked the participants to rate each robot rather than each screen display; the participants had to use the blood pressure cuff mounted half way up each robot so much of the interaction took place around the body of the robot; and participants’ responses to the characterisation questions describe aspects of the robot and not just the screen. However, future work could investigate whether these results occur when these faces are shown on computers, videos or in photographs.

## Conclusions

This work adds to the body of literature trying to discern how robot faces can affect user impressions. There are three main conclusions relating to faces on a screen display on a healthcare robot. First, the robot with the more humanlike face display was preferred to the robots with the no-face or the silver face displays, and was seen as less eerie than the robot with the silver face display. Second, people attributed more mind and more sociability to the robot with the humanlike face display than to the robot with the no-face display, and a more amiable personality to the robot with the humanlike face display than to the robots with either the silver face and no-face displays. Third, perceptions of sociability and amiability were negatively correlated with perceptions of eeriness in the robot with the humanlike face display. Robot developers need to be aware that the face they employ on a robot with a display screen will affect user impressions of the robot’s personality and mind, and that impressions of a lack of sociability and amiability may be linked to feelings of unease.
